# Improvements in Jump Height, Speed, and Quality of Life through an 8-Week Strength Program in Male Adolescents Soccer Players

**DOI:** 10.3390/sports12030067

**Published:** 2024-02-23

**Authors:** Sara Díaz-Hidalgo, Antonio Ranchal-Sanchez, Jose Manuel Jurado-Castro

**Affiliations:** 1 Ciencias De La Actividad Física y El Deporte, Escuela Universitaria de Osuna (Centro Adscrito a la Universidad de Sevilla), 41640 Osuna, Spain; saradiazh6@gmail.com; 2 Maimonides Biomedical Research Institute of Cordoba (IMIBIC), Reina Sofia University Hospital, University of Cordoba, 14004 Cordoba, Spain; 3 Department of Nursing, Pharmacology and Physiotherapy, Faculty of Medicine and Nursing, University of Cordoba, 14014 Córdoba, Spain; 4 Centro de Investigación Biomédica en Red de Fisiopatología de la Obesidad y Nutrición (CIBEROBN), Instituto de Salud Carlos III (ISCIII), 28222 Madrid, Spain

**Keywords:** male adolescents, movement velocity, soccer, exercise, physical education

## Abstract

This study aimed to assess the enhancement of physical fitness and quality of life through an 8-week strength training program in male adolescent soccer players aged between 12 and 13 years. A lower body muscle group intervention was performed, with 17 players in the experimental group (EG) and 15 players in the control group (CG). The EG carried out soccer training complemented by specific strength work. Pre- and post- intervention assessments included body composition, jump height, speed with change of direction, maximum speed in 20 m, movement velocity in back-squat, perceived fatigue effort, academic performance, and quality of life. A general linear repeated measures model analysis was used considering each variable, the interaction between groups (EG, CG) and time (basal, 8-week), to analyze the differences between and within groups. The results showed a reduction in fat in the upper limbs and trunk, improvements in jump height, maximum speed of 20 m, and changes of direction, and increased quality of life (*p* < 0.05) in the EG. It was concluded that a strength program could improve some components of physical performance in male adolescent soccer players.

## 1. Introduction

Strength training is understood as the physical conditioning method that seeks to improve a person’s ability to overcome muscle resistance [1]. When an ability is trainable, it means that it has the possibility of improvement by responding to training stimuli. In relation to strength, improvement not only depends on the conditions of the load used, but also on the characteristics of the participants [2]. According to global recommendations, children and adolescents aged 5–17 years should incorporate vigorous-intensity aerobic activities, as well as activities that strengthen muscle and bone, at least 3 days a week [3]. There is a broad international consensus supporting youth involvement in appropriately supervised strength training, endorsed by organizations such as the American Academy of Pediatrics, which emphasizes its health and training benefits [4]. Several studies demonstrate the safety and efficacy of strength training in promoting the health and performance of participants [5] and highlight the importance of strength development [2]. Conversely, soccer sports practice is characterized by its intermittent nature [6], involving repeated high-intensity, non-cyclical actions [7], such as accelerations, jumps, changes of direction, and sprints. Furthermore, there is a complementarity between strength training and soccer-specific training, potentially yielding superior results compared with a soccer-only approach. Strength training can enhance a player’s ability to execute explosive movements and sustain performance [8].

Children and adolescents who engage in team sports such as soccer may benefit from incorporating strength training into their routines, as this can enhance their physical performance [9]. Strength training has been shown to improve muscle power, a crucial physiological capacity for optimizing soccer performance [10,11,12]. In recent years, there has been an increase in the number of young individuals participating in strength training programs [1,13,14,15]. Moreover, a properly designed and implemented training regimen, incorporating specific strength exercises, can provide health benefits and enhance the development of physical conditioning and coordination, thereby improving overall performance [16]. The earlier the practice is started, the greater the improvements in the motor system and the better the adherence created in the future, making possible a better preparation for future physical activities [1,17]. In terms of general benefits, three groups stand out: musculoskeletal benefits, such as increased bone mineral density and the reduced risk of injury; benefits in overweight or obese patients, such as increased insulin sensitivity, improved blood lipid profile, and cardiovascular function; and psychosocial benefits, such as improved body image perception and increased self-esteem, self-concept, and self-perception [1]. Despite the concerns about strength training in adolescence, evidence suggests that this type of training provides benefits that extend beyond sports performance, promoting growth, maturation, and overall participant health [18]. Additionally, favorable aspects are examined in body composition, bone density, and cardiovascular functioning [2], as well as in academic performance, with observations indicating improved cognitive and sensory functioning of the brain [19] and enhanced quality of life (QoL) [20].

There is limited research focusing on the development of strength training modalities in adolescent soccer players [21], which makes widespread application in soccer challenging due to the restricted scientific literature in this area [10]. The absence of clear and concise information regarding the appropriate dosing of load volume and intensity may contribute to this challenge. Therefore, this study aimed to evaluate the improvement in physical fitness and quality of life through an 8-week targeted strength training program specifically designed for male adolescent soccer players aged between 12 and 13 years.

## 2. Materials and Methods

### 2.1. Intervention Design

An 8-week targeted strength-specific pre-post study was conducted in physically active male participants aged between 12 and 13 years. The children participated three times a week in soccer training sessions and were familiar with basic strength training exercises. There were two groups: an experimental group (EG), “Peloteros de la Sierra Sur” Football Club (Seville, Spain); and a control group (CG), “Gilena” Football Club (Seville, Spain). The intervention in the EG took place at the training center of the club. Data on the anamnesis and physical examination (injuries) of participants were reported by the participating clubs to the researchers.

**Figure 1:**
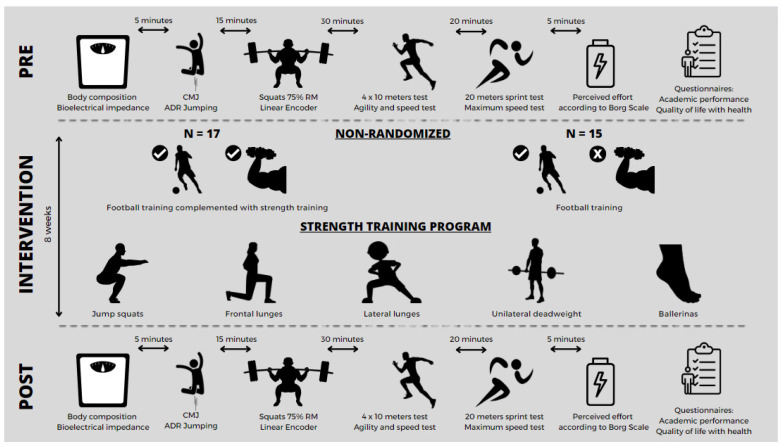
Experimental design.

At the beginning and the end of the study, participants were assessed for body composition, countermovement jumping, back squats at 75% of their one repetition maximum (RM), speed in the 4 × 10 m test, speed in the 20 m sprint test, and perceived effort according to the Borg scale. Academic performance and quality of life with health were measured by two questionnaires. These variables were taken from EG and CG (Figure 1).

The CONSORT statement was considered for the design of this study (Supplementary Material Table S1).

### 2.2. Participants

**Figure 2:**
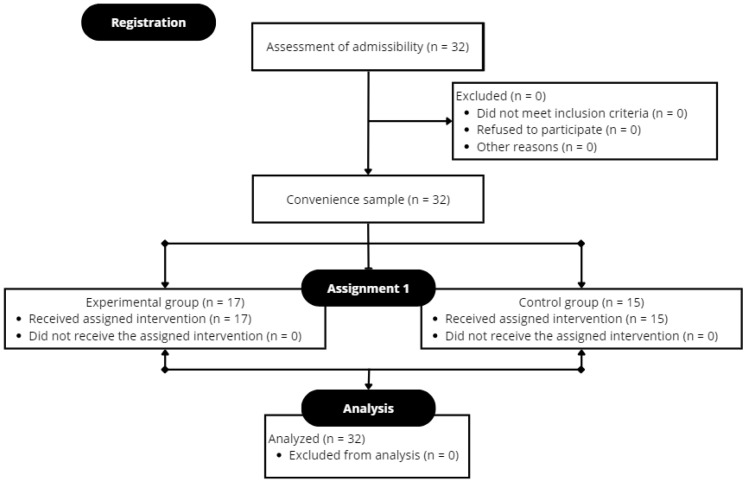
Flowchart using the CONSORT (Consolidated Standards of Reporting Trials) guidelines.

Participation was voluntary, and participants had the option to withdraw at any time; therefore, an informed consent form was signed by both the participants’ legal guardians and the sports management of both clubs. The participants recruited were briefed on the protocol and the purpose of the study and all signed a written consent prior to the start of the research. The study was conducted in accordance with the Declaration of Helsinki (52nd General Assembly Edinburgh, Scotland, October 2000). All participants completed the trial (Figure 2).

#### Sample Collection Criteria

For the selection of the sample, inclusion and exclusion criteria were employed to ensure that all participants had equal conditions. In the case of the inclusion criteria, the following were required: I. Be between 12 and 13 years of age at the initial measurement; II. Have been training in soccer for more than one year; III. Possess some familiarity with basic strength training exercises; IV. Lack musculoskeletal injuries that could interfere with the exercise program to be carried out during the research. As for the exclusion criteria, the following were highlighted: I. Failure to attend more than two strength training sessions; II. Experience of an injury or an inadequate state of health during the study intervention period, hindering the correct execution of the training program; III. Have any medical condition that contraindicates engaging in physical exercise.

All players underwent medical examinations to confirm their health status. Additionally, the trainers were instructed to report any issues that arose during the training sessions. No adverse effects were reported, and there were no instances of participant absenteeism during the study period.

### 2.3. Intervention

The intervention was carried out in the EG, who performed soccer training complemented with strength training; while participants in the CG who did not perform the intervention program, but did perform the soccer training, participated in the intervention. The strength intervention lasted for 8 weeks. The strength training program consisted of 16 training sessions, carried out with a frequency of two sessions per week. This training consisted of five exercises, targeting five different specific muscle groups of the lower body: calf muscles, quadriceps, hamstrings, abductors, and adductors. Three sets of 12 repetitions each were performed, with a recovery between sets of 2 min and a medium movement velocity.

The strength program was supervised through a direct observation by a specialist technician and was performed before each soccer training session. The exercises performed were squats with jumping, frontal lunges, lateral lunges, unilateral dead weights, and ballerinas. The specific soccer training was carried out 3 days a week, with a duration of 120 min per day. All training sessions were held in the afternoon, always complying with the established schedule. The soccer training was based on technical–tactical aspects and was the same for both teams, as well, both the EG and the CG had the same training frequency. The volume of soccer-specific work was similar in both groups, with exercises focused on pressure after loss, progression, ball circulation, and field occupation, among others.

The external load used by some participants was calculated and added according to their RM [22]; this load was established according to the familiarity with strength training and high sports performance shown to their coaches in soccer. On the other hand, we had the help of the club, who had previous records of those participants who require greater stimulation thanks to interventions carried out previously. Finally, questionnaires were completed thanks to the participants’ family members, who provided information on academic results [23], while the participants reflected on the frequency with which they performed certain actions related to QoL and health [20].

Despite being different clubs, the sample size did not allow the use of a single team. The coaches provided researchers with a report after each training session. These reports detailed the physical content covered, the health status of the players, the perceived group dynamics, the level of interest shown, and the attendance of all participants.

### 2.4. Variables

Body composition with bioimpedance

Anthropometric measurements and body composition of all participants were recorded during the first and last visits. Bioelectrical impedance (Tanita MC-780MA; Tanita Corporation, Tokyo, Japan) was measured to report body composition, with a special emphasis on body fat percentage and body mass index (BMI). The measurement was performed according to the protocol of The International Society for the Advancement of Kinanthropometry (ISAK). Height was measured using a stadiometer (Seca 214 portable stadiometer; Seca, Hamburg Germany). The participant stood barefoot, upright, and facing the horizon.

Muscular power in countermovement jumping (CMJ)

A warm-up consisting of three CMJs at a moderate intensity (60–70% of perceived maximum performance) was performed. Subsequently, with 2 min rest between them, the participants were instructed to start with an initial upright position and descend by bending their knees at a 90 degree angle, keeping their hands on their hips with their trunk upright and taking care not to interrupt the movement from the beginning of the jump to the end. Three CMJs were performed, with a recovery period of 45 s between jumps, observed by an evaluator who stood at a distance of 1.5 m in the frontal plane to control the correct execution of the jump and record the maximum height (cm) reached in the three attempts [24]. The height reached was recorded using an infrared measurement sensor (ADR Jumping, Ciudad Real, Spain).

Movement velocity and power in back-squats at 75% RM

In order to guarantee the participant’s maximum performance while eliminating possible muscle fatigue, it is important to note that all back-squats were performed the day after the rest of the tests. For the warm-up, back-squats were performed with a free barbell weighing 15 kg. Subsequently, the load was increased based on their RM. To achieve this, the maximum explosive speed was used for a total of three repetitions. The participant’s technique was assessed, and corrections were made when necessary. To ensure that the technique was considered satisfactory, participants stood with their feet shoulder-width apart and the bar resting on their shoulder blades, while their hands gripped on the bar. Afterwards, they flexed their knees to 90 degrees, and subsequently, they performed the extension back to the original standing position [25]. The primary load for all participants was 15 kg and was gradually increased by 2.5 kg until an average velocity of 0.86 m/s was reached. Subsequently, the load was adjusted in smaller increments (1.25 kg) for each participant, to calculate 75% 1 RM accurately, when the velocity reached 0.76 m/s. To ensure safe weightlifting, there were well-trained volunteers on both sides of the bar. The rest between sets was 3 min. Only the fastest average speed repetition was considered for the analysis. The velocity was controlled by a linear position transducer (encoder) (Speed4Lift v.4.1, Speed4Lift, Madrid, Spain), employed previously [26].

Back-squats to failure at 75% RM

After 20 min of active rest, three repetitions with a free bar (15 kg) were performed for a new warm-up. Subsequently, the weight set on the previous day for each participant was set at 75%; with this weight, the participants performed the maximum number of back-squats possible. It is important to note that there were qualified technicians intervening and assisting in the performance of this test, to ensure the safety of the adolescents at all times. This test was performed twice, with a 5-min rest per participant. The highest number of repetitions that each participant was able to perform was recorded.

4 × 10 m test

A brief dynamic warm-up lasting 5 min was conducted to improve flexibility and prevent injuries. The warm-up consisted of continuous running, stretching, joint mobility exercises, and multiple displacements, varying the intensity. Next, the test was extracted from the ALPHA-Fitness battery [27,28]. Two parallel lines were established at a distance of 10 m. In this line, five marks were highlighted: (a) start mark; (b) first sponge mark; (c) second sponge mark; (d) third sponge mark; and (e) finish mark. The objective of this test focuses on moving through the five marks at maximum speed and in the correctly established order. The sponge at mark b should end at mark c; the sponge at mark c should end at mark d, and the sponge at mark d should end at the finish mark. The measurement was made with a stopwatch (seconds), two measurements were made and the best result was recorded. The maximum speed when running and turning in a delimited area was recorded, it could be performed twice. The best repetition was selected for further analysis.

20 m sprint test

After 15 min of active rest, the test was performed to measure the reaction speed and acceleration possessed over 20 m [2]. The participant waited for the start sample and, after that, they ran at maximum speed up to the established mark at a total distance of 20 m. To perform this test, it is required to delimit the starting line and the final line of 20 m. The measurement was made with a stopwatch (seconds), two measurements were taken and the best result was recorded. The maximum reaction and acceleration speed over a distance of 20 m was recorded. Participants’ perceived fatigue effort during the assessment.

Perceived effort

At the end of each evaluation, the participants’ perception of effort was measured using the Borg scale, offering a score from 1 to 10. The perceived effort after each workout performed was also evaluated [29].

Academic results

It was analyzed whether the practice of collective sports complemented with strength training improved academic results, by means of an ad hoc questionnaire (Supplementary Material Table S2) completed by the parents of the participants when they were minors. In addition, the grades of the first trimester and those of the second trimester were assessed to analyze whether the academic results were modified after sports practice, based on the instrument used previously [23]. The mentioned questionnaire is similar to the one used in a scientific article where each subject is valued as insufficient, sufficient, good, notable, and outstanding [30].

Health-related quality of life

The health-related QoL of the participants was assessed using a questionnaire, Kiddo-Kindl [31], before and after the intervention (Supplementary Material Table S3). The reliability index was 0.87 with a factorial structure of six dimensions: emotional well-being, self-esteem, school welfare and interest, fun with peers, family well-being, and physical well-being; as conducted in a study, where the validity and reliability of the instrument was guaranteed worldwide and cross-culturally, as it was suitable for measuring QoL [20]. This questionnaire was completed by the participants at home, under the supervision of their legal guardian, with the aim of analyzing whether sports practice with a strength intervention favors the participants’ QoL.

### 2.5. Sample Size

As the RM on strength exercise was one of the main outcomes of this study, the sample size was determined by calculating the statistical power based on a previous study [2], with a power of 0.80 and a two-tailed α level set to 0.05; the minimum number of participants required to detect a 10% difference in RM was estimated as 32.

### 2.6. Statistical Analysis

SPSS software (Version 29.0, IBM SPSS for Windows, 2022) was used to examine the data, obtaining descriptive statistics such as the mean and standard deviation of each variable investigated. Reliability was assessed using intraclass correlation coefficients and coefficients of variation. The homogeneity of variance of the EG and CG groups was examined using Levene’s test, and the normality of the data was assessed using the Shapiro–Wilk test. A general linear repeated measures model analysis was used considering each variable [32]; the interaction between groups (EG, CG) and time (Basal, 8-week) was used to analyze the differences between groups and within groups. Repeated measures test effect size was calculated using partial eta squared (η^2^p), with a small effect size considered below 0.25, medium as 0.26–0.63, and large above 0.63. Statistical significance was set at *p* < 0.05 for all inference tests [33].

## 3. Results

The characteristics and body composition of the participants at baseline and the 8-week visit (both EG and CG) are shown in Table 1. Although there was an increase in height due to growth, there were no improvements in weight, percentage of fat, and BMI in the time×group interaction, neither in the EG nor in the CG. However, a reduction in the percentage of fat in the upper extremities and trunk was observed in the EG. As for the lower extremities, no improvements were observed in any group or during the time that elapsed between the baseline and the 8-week measurements (Table 1).

The EG obtained improvements in the CMJ after the period of time between the baseline and the 8-week measurement. The intervention seemed to have an effect since improvements were observed in the time×group interaction (Table 2).

The RM in back-squats, the MV and MP in back-squats at 75% of RM, and back-squats to failure, improvements were examined between baseline and the end of the intervention program in both groups (Table 2).

The 4 × 10 m test and the 20 m sprint test showed improvements after the strength intervention, as shown in Table 2. The 4 × 10 m test showed improvements in both teams after the time period between the baseline and the 8-week measurements; thus, the intervention caused a significant effect, as improvements were shown in the time*group interaction (Table 2).

The 20 m sprint test showed improvements between the baseline and the 8-week measurements only in the EG; however, the CG did not obtain relevant improvements. However, the intervention had a significant effect, since improvements were observed in the time*group interaction (Table 2).

In perceived fatigue effort, measured by the Borg scale, effect for time was reported (*p* = 0.002; η^2^p = 0.521), but neither reported any effect for group (*p* = 0.847; η^2^p = 0.003) or time×group (*p* = 0.922; η^2^p = 0.001).

In the academic results, improvements were observed for time (*p* = 0.006; η^2^p = 0.428) and for group (*p* = 0.020; η^2^p = 0.328); however, no improvements were found in the interaction time×group (*p* = 0.540; η^2^p = 0.027).

Health-related QoL, in general, showed improvements in both groups between the baseline and 8-week measurements; in addition, the intervention had a significant effect as improvements were observed in the time×group interaction (Table 3). It was observed that self-esteem obtained improvements between the baseline and the 8-week measurements in the EG; improvements were even observed in the time×group interaction, so the strength intervention produced a significant effect (Table 3). Emotional and school well-being only obtained improvements in relation to baseline and 8-week measurements in both groups (Table 3). The relationship with friends showed improvements between groups, where EG was superior to CG (Table 3). Moreover, in Table 3, physical well-being showed improvements between the baseline and 8-week measurements in the EG, but not in the CG.

## 4. Discussion

The aim of this study was to assess the enhancement of physical fitness and quality of life in an 8-week strength training program comparing two groups of Spanish male adolescent soccer players aged between 12 and 13 years. The main findings show that the EG experienced fat reduction in their upper extremities and trunk, and improvements in the fitness variables CMJ, the 4 × 10 m test, and the 20 m sprint test, as well as in health-related QoL.

A well-designed strength training program can be considered as a safe strategy to improve this physical quality in participants [34]. The main findings of this study suggest that the practice of soccer supplemented with a strength training program for 8 weeks, with a frequency of 2 days per week and a volume of five exercises with three sets, each of 12 repetitions and a rest of 2 min, produces improvements in the sports performance and physical fitness of adolescent soccer players.

Several studies [35,36,37,38] affirm that strength training in soccer players is likely to be adequate to achieve interesting results on strength in their sports performance [39].

In this study, it could be observed that a frequency of 2 days per week of strength training could be sufficient to introduce adolescent players to strength development. Therefore, these results could be translational because the study was performed in compulsory schooling individuals who have physical education classes with a frequency of two sessions per week throughout the academic year.

Similarly, other studies use the same frequency with a similar prototype population, achieving improvements in this physical capacity [2,40,41]. On the contrary, reducing the work to 1 week would minimize the effect of the program on strength gain [36].

In this study, although no influences on BMI were found, there were improvements in fat reduction in the upper extremities and trunk in the EG. Following this, a review [42] reported positive changes in body composition without improvements in BMI. Puberty is a stage of continuous physiological changes and growing up, which has an impact on the development of physical abilities [18]. In relation to the EG improvement of physical condition in the trunk and upper extremities, it is believed that this was due to the duration of the program, since it was 8 weeks, a period stipulated to obtain improvements according to several studies [35,36,38]. Therefore, the improvements obtained in our participants could be due to the effect of the strength training program and not only to physiological reasons related to their growth, with the exception of age. Therefore, the longer and quicker growth of legs could explain why differences were found in fat reduction only in the adolescents’ upper extremities. Furthermore, during the strength program, the EG participants included external loads that favored the upper body, since they involved an external stimulus to which their bodies were not accustomed. However, the lower train of the participants did not obtain improvements in fat reduction; this could also be due to the continuous development of these extremities in sports such as soccer, which implies much more delayed effects in the lower train than in the upper train [43].

One interesting aspect is the improvement in the CMJ variable, because of the desirable jumping ability in some actions of soccer players, such as “head a ball” or the goalkeeper functions. In this study, it was possible to verify the improvement obtained in the CMJ variable through the strength training program in adolescent soccer players, who increased their jump height and their flight time. This same conclusion was obtained in a similar study with a similar sample, where a strength and plyometric training program was proposed [44].

Regarding other physical fitness qualities, the progress of several skills in collective sports such as soccer can be influenced by a properly developed physical quality [45]. Running speed, reaction speed, agility, and changes in direction are characteristics of the sport in question and some studies with similar samples claim a transfer from strength training to speed over 30 m [8,46]. Similarly, the results of this study showed a beneficial relationship between strength development and running time in tests such as the 20 m sprint test and the 4 × 10 m test [47].

The competitive period in which the participants found themselves is characterized by a high technical–tactical training load, which is an issue that could affect sports performance in variables such as back-squats. In addition, knee flexion angle and execution technique could have influenced the results, as no significant interaction was observed after the intervention [48].

No improvements were observed in the perception of effort after the intervention, which could be related to external factors not considered in this study (like the puberty hormonal release) that could affect this variable. Furthermore, it could be possible that there was a relationship between emotional state, cognitive perceptions, and physical state, which influenced the subjective perception of the individual effort [49].

Regarding academic performance, it is important to note that the EG already had a high academic average, so further increasing their performance is a complicated aspect. Although the practice of physical activity is related to obtaining good academic results [50], there is no evidence to affirm that strength training did not seem to increase the academic performance in our study.

Finally, QoL (overall and self-esteem) is one of the variables that demonstrated improvements after the practice of soccer and strength training in this study. Therefore, it is important to highlight the intervention by the World Health Organization who affirm these results, since they defend that physical activity improves mood and decreases the risk of suffering from stress, anxiety, and depression, increasing self-esteem and providing greater psychological well-being. Nowadays, emotional aspects in sports are becoming more and more relevant. The improvement in the EG overall and the self-esteem QoL shows that this strength program would be good to enhance it.

The primary limitation is the non-random sampling method employed. Additionally, factors such as maturity status (puberty stages), lean body (muscle) mass or appendicular lean mass, habitual physical activity levels of the participants, and involvement in other organized sports activities during the intervention period were not assessed. The results of this study should be interpreted with caution.

On the contrary, the study has the strength to constitute a practical and feasible intervention that could be translocated to the academic and competitive sectors.

## 5. Conclusions

A strength program could improve some components of physical performance in male adolescent soccer players, such as jump height, maximum speed of 20 m, and changes of direction, reducing the fat in the uppers limbs and trunk, as well as an increment in the overall and self-esteem QoL.

These findings may have practical applicability in sports performance and the physical conditions involved in competitive soccer practice. The improvement of the variables studied, especially those related to the power of the lower body, have a direct transfer in the aerial game of both the field players and the goalkeeper in the practice of soccer.

## Figures and Tables

**Table 1:** Descriptive characteristics and comparative analysis of averages for measurements in body composition of both groups at the beginning and the end of the strength training program.

Variable	Group	Baseline (0-Week)	Final (8-Week)	*p* Value Time	η^2^p	*p* Value Group	η^2^p	*p* Value Time × Group	η^2^p
**Age** **(years)**	EG	12.5 ± 0.5	12.7 ± 0.6	0.004 *	0.467	0.265	0.088	0.719	0.010
	CG	12.7 ± 0.5	12.9 ± 0.7						
**Height** **(cm)**	EG	154.9 ± 7.9	155.1 ± 8	0.024 *	0.313	0.012 *	0.373	0.181	0.124
	CG	161.4 ± 6.3	162.5 ± 6.7						
**Weight** **(kg)**	EG	43.5 ± 7.6	44 ± 7.8	0.003 *	0.475	0.063	0.226	0.356	0.061
	CG	50.9 ± 12	51.8 ± 12.1						
**BMI** **(kg/m^2^)**	EG	18 ± 2.1	18.2 ± 2.1	0.261	0.089	0.327	0.069	0.597	0.021
	CG	19.4 ± 4.1	19.5 ± 4.1						
**Fat** **(%)**	EG	18.5 ± 2.7	17.3 ± 2.7	0.027 *	0.304	0.672	0.013	0.139	0.149
	CG	18.6 ± 4	18.3 ± 4.3						
**RA-Fat** **(%)**	EG	30.2 ± 4.1	28.5 ± 3.4	0.023 *	0.319	0.050 *	0.248	0.025 *	0.310
	CG	27.4 ± 4.5	26.8 ± 3.8						
**LA-Fat** **(%)**	EG	30 ± 3.3	28 ± 3.4	0.002 *	0.518	0.382	0.055	0.043 *	0.261
	CG	28.1 ± 4	27.7 ± 4.2						
**TR-Fat** **(%)**	EG	15.2 ± 3.1	14 ± 3.1	0.105	0.177	0.824	0.004	0.034 *	0.284
	CG	14.8 ± 4.6	15.1 ± 4.9						
**RL-Fat** **(%)**	EG	19.7 ± 2.8	19 ± 2.7	0.104	0.177	0.373	0.057	0.257	0.091
	CG	20.5 ± 4.1	20.4 ± 4.4						
**LL-Fat** **(%)**	EG	20.4 ± 2.8	19.6 ± 2.7	0.205	0.112	0.351	0.062	0.149	0.143
	CG	21.2 ± 4.3	21.3 ± 4.7						

Note. * Statistically significant difference, *p* < 0.05. Data expressed as mean ± standard deviation. η^2^p: effect size was calculated using partial eta squared; RA: right arm; LA: left arm; CG: control group; EG: experimental group; BMI: body mass index; RL: right leg; LL: left leg; TR: trunk.

**Table 2:** Descriptive characteristics and comparative analysis of averages for measurements in RM, CMJ, 4 × 10 m test, and 20 m sprint test in both groups, at the beginning and the end of the strength training program.

Variable	Group	Baseline (0-Week)	Final (8-Week)	*p* Value Time	η^2^p	*p* Value Group	η^2^p	*p* Value Time × Group	η^2^p
**CMJ** **(cm)**	EG	34.7 ± 4.3	36.1 ± 3.7	0.006 *	0.430	0.385	0.054	0.007 *	0.415
	CG	34 ± 3.9	34 ± 3.6						
**Back-Squat RM** **(kg)**	EG	33.3 ± 6.4	38.2 ± 6.8	0.006 *	0.424	0.002 *	0.496	0.086	0.196
	CG	42.7 ± 5.7	44.3 ± 7.7						
**75%** **Back-Squat MV** **(m/s)**	EG	0.73 ± 0.06	0.68 ± 0.04	<0.001 *	0.611	0.044 *	0.260	0.588	0.021
	CG	0.7 ± 0.04	0.66 ± 0.03						
**75%** **BACK-SQUAT PV** **(m/s)**	EG	1.06 ± 0.06	2.32 ± 3.3	0.164	0.134	0.135	0.153	0.155	0.139
	CG	1.05 ± 0.09	1.03 ± 0.09						
**75%** **Back-Squat MP** **(w)**	EG	311.9 ± 60.2	294.7 ± 55	0.001 *	0.540	0.186	0.122	0.662	0.014
	CG	349 ± 85.6	335 ± 79.3						
**75%** **Back-Squat PP** **(w)**	EG	452.4 ± 78	921 ± 1203	0.159	0.136	0.303	0.076	0.158	0.137
	CG	523.4 ± 129.5	525.2 ± 127						
**Back-Squat to failure (Number of repetitions)**	EG	7.6 ± 1	8.1 ± 0.9	0.010 *	0.388	0.436	0.044	0.582	0.022
	CG	7.2 ± 1	7.8 ± 1.1						
**4 × 10 m test** **(seg)**	EG	10.67 ± 0.3	10.42 ± 0.3	<0.001 *	0.808	<0.001 *	0.617	<0.001 *	0.743
	CG	10.06 ± 0.2	10.06 ± 0.2						
**20 m sprint test** **(seg)**	EG	3.84 ± 0.15	3.72 ± 0.14	<0.001 *	0.691	0.939	0.000	0.003 *	0.471
	CG	3.79 ± 0.12	3.77 ± 0.15						

Note. * Statistically significant difference, *p* < 0.05. Data are expressed as mean ± standard deviation. η^2^p: effect size was calculated using partial eta squared; CMJ: countermovement jump; CG: control group; EG: experimental group; MP: mean power; PP: peak power; RM: repetition maximum; MV: mean velocity; PV: peak velocity.

**Table 3:** Descriptive characteristics and comparative analysis of averages for the measurements of health-related quality of life in both groups, at the beginning and the end of the strength training program.

Variable	Group	Baseline (0-Week)	Final (8-Week)	*p* Value Time	η^2^p	*p* Value Group	η^2^p	*p* Value Time × Group	η^2^p
**QoL emotional well-being**	EG	4.3 ± 0.6	4.5 ± 0.5	0.002 *	0.520	0.020 *	0.328	0.841	0.003
	CG	3.8 ± 0.6	3.9 ± 0.4						
**QoL self-esteem**	EG	4.2 ± 0.7	4.6 ± 0.4	0.003 *	0.472	0.237	0.098	0.014 *	0.360
	CG	4.1 ± 0.7	4.1 ± 0.5						
**QoL school**	EG	4.1 ± 0.8	4.6 ± 0.4	0.049 *	0.250	0.030 *	0.293	0.063	0.225
	CG	3.5 ± 1.3	3.6 ± 1.2						
**QoL friends**	EG	4.7 ± 0.2	4.8 ± 0.3	0.282	0.082	0.048 *	0.252	0.774	0.006
	CG	4.4 ± 0.5	4.5 ± 0.4						
**QoL family well-being**	EG	4.6 ± 0.3	4.7 ± 0.3	0.132	0.154	0.081	0.201	0.845	0.003
	CG	4.3 ± 0.5	4.4 ± 0.4						
**QoL physical well-being**	EG	3.7 ± 0.6	4.4 ± 0.4	<0.001 *	0.711	0.493	0.034	0.105	0.176
	CG	3.7 ± 0.4	4.1 ± 0.5						
**Overall quality of life**	EG	4.3 ± 0.4	4.6 ± 0.2	<0.001 *	0.783	0.004 *	0.464	0.018 *	0.340
	CG	4 ± 0.3	4.1 ± 0.3						

Note. * Statistically significant difference, *p* < 0.05. Data are expressed as mean ± standard deviation. η^2^p: effect size was calculated using partial eta squared; CG: control group; EG: experimental group; QoL: quality of life.

## Data Availability

Data are contained within the article and Supplementary Materials.

## Supplementary Materials

The following supporting information can be downloaded at: https://www.mdpi.com/article/10.3390/sports12030067/s1, Table S1: CONSORT 2010. Checklist of information to include when reporting a randomized clinical trial. Table S2: Academic results questionnaire. Table S3: Academic results questionnaire.
