# Supplementary material for: Improvements in Jump Height, Speed, and Quality of Life through an 8-Week Strength Program in Male Adolescents Soccer Players
Source: Sports (Basel). 2024 Feb 23;12(3):67. doi: 10.3390/sports12030067 (PMC10975651; doi:10.3390/sports12030067)
Supplement: Supplementary file 1 [file sports-12-00067-s001.zip › sports-2864539-supplementary.pdf]

**Table S1.** CONSORT 2010. Checklist of information to include when reporting a randomized clinical trial.

| Section/Topic | Item No. | Checklist item | Reported on page No. |
|---|---|---|---|
| **Title and summary** | | | |
| | 1a | Identified as a randomized trial in the title. | p 1 |
| | 1b | Structured summary of the trial design, methods, results and conclusions. | p 1 |
| **Introduction Background and objectives** | | | |
| | 2a | Scientific basis and explanation of the reasons | p 1 and 2 |
| | 2b | Specific objectives or hypotheses | - |
| **Methods** | | | |
| **Trial design** | 3a | Description of the trial design (e.g., parallel, factorial), including allocation proportion | p 2 and 3 |
| | 3b | Significant changes in methods after trial initiation (such as eligibility criteria), with reasons | - |
| **Participants** | 4a | Criteria for selection of participants | p 3 and 4 |
| | 4b | Environments and data collection sites | p 4 |
| **Interventions** | 5 | Interventions for each group with sufficient detail to allow for replication, including how and when they were actually administered | p 4 - 6 |
| **Results** | 6a | Fully defined primary and secondary outcome measures, including how and when they were assessed | p 7 - 10 |
| | 6b | Any change in the results of the trial after the start of the trial, with reasons for the change. | - |
| **Sample size** | 7a | How the sample size was determined | p 6 |
| | 7b | Where appropriate, explanation of interim analysis and discontinuation guidelines | - |
| **Randomization** | | | |
| **Sequence generation** | 8a | Method used to generate the randomization sequence | - |
| | 8b | Type of randomization; details of any constraints (such as blocks and block size) | - |
| **Allocation concealment mechanism** | 9 | Mechanism used to implement the randomization sequence (such as sequentially numbered garbage cans), describing any measures taken to hide the sequence until the interventions were allocated | - |
| **Implementation** | 10 | Who generated the randomization sequence, who enrolled the participants and who assigned them to the interventions. | - |
| **Masking** | 11a | If so, who was blinded after assignment to the interventions (e.g., participants, health professionals, those assessing outcomes) and how? | - |
| | 11b | If applicable, description of the similarity of the interventions | - |

| | | | |
|---|---|---|---|
| **Statistical methods** | 12a | Statistical methods used to compare primary and secondary outcome groups | p 6 |
| | 12b | Methods for additional analyses, such as subgroup analysis and adjusted analysis. | - |
| **Results** | | | |
| **Participant flow (a flow chart is strongly recommended)** | 13a | For each group, the number of participants who were randomly assigned, received the intended treatment, and were analyzed for the primary outcome. | p 3 |
| | 13b | For each group, the losses and exclusions after randomization, together with the reasons | p 3 |
| **Recruitment** | 14a | Dates defining recruitment and follow-up periods | p 2 |
| | 14b | Why the trial was terminated or interrupted | - |
| **Baseline data** | 15 | A table showing demographic and clinical baseline characteristics for each group | - |
| **Numbers analyzed** | 16 | For each group, number of participants (denominator) included in each analysis and whether the analysis was based on the initially assigned groups. | p 3 |
| **Results and estimation** | 17a | For each primary and secondary outcome, the results for each group and the estimated effect size and precision (as the 95% confidence interval). | p 7 - 11 |
| | 17b | For binary results, the presentation of both absolute and relative effect sizes is recommended. | p 7 - 11 |
| **Secondary analysis** | 18 | Results of any other analyses performed, including subgroup analyses and adjusted analyses, distinguishing pre-specified from exploratory analyses. | - |
| **Damages** | 19 | All significant damages or undesirable effects in each group | - |
| **Discussion** | | | |
| **Limitations** | 20 | Trial limitations, addressing sources of potential bias, imprecision, and, if relevant, multiplicity of analyses | p 11 and 12 |
| **Generalization** | 21 | Generalizability (external validity, applicability) of trial findings | p 12 |
| **Interpretation** | 22 | Interpretation consistent with the results, with balance of benefits and harms, and considering other relevant evidence. | |
| **Other information** | | | |
| **Registration** | 23 | Registration number and name of the trial registration | - |
| **Protocol** | 24 | Where to find the full trial protocol, if available | - |
| **Financing** | 25 | Sources of financing and other support (such as drug supply), role of funders | - |

*Note: own elaboration extracted from CONSORT 2010.*

**Table S2.** Academic results questionnaire.

| **Soccer player's name:** | | | | |
|---|---|---|---|---|
| **Team to which he belongs:** | | | | |
| Peloteros de la Sierra Sur Sports Club | | Gilena Football Club | | |
| **Spanish Language and Literature** | | | | |
| Insufficient | Sufficient | Well | Notable | Outstanding |
| **Mathematics** | | | | |
| Insufficient | Sufficient | Well | Notable | Outstanding |
| **Geography and History** | | | | |
| Insufficient | Sufficient | Well | Notable | Outstanding |
| **Physical Education** | | | | |
| Insufficient | Sufficient | Well | Notable | Outstanding |
| **Catholic Religion / Ethical Values** | | | | |
| Insufficient | Sufficient | Well | Notable | Outstanding |
| **Biology and Geology** | | | | |
| Insufficient | Sufficient | Well | Notable | Outstanding |
| **French / Elective** | | | | |
| Insufficient | Sufficient | Well | Notable | Outstanding |
| **English** | | | | |
| Insufficient | Sufficient | Well | Notable | Outstanding |
| **Music** | | | | |
| Insufficient | Sufficient | Well | Notable | Outstanding |
| **Public Speaking and Debate** | | | | |
| Insufficient | Sufficient | Well | Notable | Outstanding |

| Tutoring | | | | |
|---|---|---|---|---|
| Insufficient | Sufficient | Well | Notable | Outstanding |
| **In case you have taken any subject not mentioned in the questionnaire, please indicate it together with the grade obtained:** | | | | |

Questionnaire extracted from an article [23]; furthermore, it is similar to the one used in a scientific article [30]

**Table S3.** Health-related quality of life questionnaire.

| **Soccer player's name:** | | | | |
|---|---|---|---|---|
| **Team to which he belongs:** | | | | |
| Peloteros de la Sierra Sur Sports Club | | Gilena Football Club | | |
| **I have felt lonely.** | | | | |
| Never | Almost never | Sometimes | Almost always | Always |
| **I have been bored out of my mind.** | | | | |
| Never | Almost never | Sometimes | Almost always | Always |
| **I felt different from the others.** | | | | |
| Never | Almost never | Sometimes | Almost always | Always |
| **I have felt afraid or insecure.** | | | | |
| Never | Almost never | Sometimes | Almost always | Always |
| **I have felt burdened by my parents.** | | | | |
| Never | Almost never | Sometimes | Almost always | Always |

<table>
<tr><td colspan="5">I liked myself.</td></tr>
<tr><td>Never</td><td>Almost never</td><td>Sometimes</td><td>Almost always</td><td>Always</td></tr>
<tr><td colspan="5">I have been proud of myself.</td></tr>
<tr><td>Never</td><td>Almost never</td><td>Sometimes</td><td>Almost always</td><td>Always</td></tr>
<tr><td colspan="5">I felt good about myself.</td></tr>
<tr><td>Never</td><td>Almost never</td><td>Sometimes</td><td>Almost always</td><td>Always</td></tr>
<tr><td colspan="5">I have been interested in the classes.</td></tr>
<tr><td>Never</td><td>Almost never</td><td>Sometimes</td><td>Almost always</td><td>Always</td></tr>
<tr><td colspan="5">I have been able to do my homework well.</td></tr>
<tr><td>Never</td><td>Almost never</td><td>Sometimes</td><td>Almost always</td><td>Always</td></tr>
<tr><td colspan="5">I have had many good ideas.</td></tr>
<tr><td>Never</td><td>Almost never</td><td>Sometimes</td><td>Almost always</td><td>Always</td></tr>
<tr><td colspan="5">I got along well with my friends.</td></tr>
<tr><td>Never</td><td>Almost never</td><td>Sometimes</td><td>Almost always</td><td>Always</td></tr>
<tr><td colspan="5">I laughed and had a lot of fun.</td></tr>
<tr><td>Never</td><td>Almost never</td><td>Sometimes</td><td>Almost always</td><td>Always</td></tr>
<tr><td colspan="5">The others liked me.</td></tr>
<tr><td>Never</td><td>Almost never</td><td>Sometimes</td><td>Almost always</td><td>Always</td></tr>
<tr><td colspan="5">I have had a lot of strength and energy.</td></tr>
<tr><td>Never</td><td>Almost never</td><td>Sometimes</td><td>Almost always</td><td>Always</td></tr>
<tr><td colspan="5">I got along well with my parents.</td></tr>
<tr><td>Never</td><td>Almost never</td><td>Sometimes</td><td>Almost always</td><td>Always</td></tr>
<tr><td colspan="5">I have felt at home.</td></tr>
<tr><td>Never</td><td>Almost never</td><td>Sometimes</td><td>Almost always</td><td>Always</td></tr>
</table>

| | | | | |
|---|---|---|---|---|
| **We have had arguments or fights at home.** | | | | |
| Never | Almost never | Sometimes | Almost always | Always |
| **I felt sick.** | | | | |
| Never | Almost never | Sometimes | Almost always | Always |
| **It hurt a little.** | | | | |
| Never | Almost never | Sometimes | Almost always | Always |
| **I have been very tired and exhausted.** | | | | |
| Never | Almost never | Sometimes | Almost always | Always |

Kiddo-Kindl questionnaire extracted from an article [31].
